# How weather conditions affect well-being: an explanation from the perspective of environmental psychology

**DOI:** 10.3389/fpubh.2025.1553315

**Published:** 2025-07-04

**Authors:** Weida Zhang, Wenzhang Li

**Affiliations:** ^1^School of Education, Huaibei Normal University, Huaibei, Anhui, China; ^2^School of Education, Jiangxi Normal University, Nanchang, China

**Keywords:** environmental psychology, weather conditions, well-being, empirical evidence, physiological mechanisms

## Abstract

Currently, the issue of how weather conditions influence well-being has garnered widespread attention from researchers, and the rise of environmental psychology has provided a novel theoretical framework and quantitative analytical tools for understanding the relationship between the two. Through a review of domestic and international literature, this paper reveals the complex and interactive relationship between weather conditions and well-being. Firstly, weather conditions, as an important component of the natural environment, encompass multiple dimensions such as light, temperature, precipitation, humidity, wind speed, air quality, and extreme weather events. These factors not only directly influence various dimensions of well-being but may also interact to form cumulative effects that collectively shape an individual’s well-being experience. Secondly, weather conditions indirectly affect well-being levels by influencing an individual’s physiological state, emotional mood, and patterns of social interaction. This paper provides a thorough review of the theoretical basis, empirical evidence, and physiological mechanisms of how weather impacts well-being, offering theoretical references for enhancing individual well-being and suggesting directions for future research.

## 1 Introduction

Well-being is not only the core pursuit of human life but also an important predictor of various positive life outcomes, such as longevity, creativity, quality of interpersonal relationships, and work efficiency (1). In daily life, individuals often closely associate their well-being with weather conditions. For example, good weather is commonly believed to enhance well-being, while harsh weather is usually associated with negative emotions and low well-being. Everyday experience suggests that sunny weather is often accompanied by a pleasant mood, whereas cold, gloomy, or cloudy weather tends to trigger negative emotions and unpleasant experiences. In ancient Chinese literature, the relationship between weather conditions and well-being is particularly evident. In recent years, with the deepening of natural science research, scholars have started to explore the relationship between weather conditions and well-being in a more systematic manner. This paper uses the Web of Science database as the data source, with a search date of December 21, 2023. The keywords “weather,” “weather condition,” and “temperature” were searched in combination with terms such as “well-being,” “subjective well-being,” “happiness,” and “emotion.” The literature search was limited to publications between 1900 and 2023, and a total of 91 relevant articles were obtained. A statistical analysis of the frequency of publication in core journals shows a significant increase in research on the relationship between weather conditions and well-being in recent years, especially in the past 5 years, when research output rapidly expanded. This trend reflects growing academic attention to the field and the deepening exploration of its theoretical depth. The research trend indicates that the relationship between weather conditions and well-being is not only a practical and meaningful research topic but also a complex phenomenon worthy of in-depth discussion from multidisciplinary perspectives. By systematically reviewing existing literature, we can gain a clearer understanding of how weather factors influence individual well-being through physiological, psychological, and social interaction pathways, thus providing a reference for theoretical development and policy-making in related fields.

Environmental psychology, as an important branch of psychology and a discipline at the intersection of psychology and environmental science, provides an effective “dialogue” point for the subjectivity of psychology and the objectivity of the environment in quantitative research. With the development of the field, environmental psychology has gradually become an important theoretical framework for understanding human emotions, cognition, and their relationships with the environment. In measuring well-being, psychology typically examines two key dimensions: emotion, which includes positive and negative affect, and cognition, which pertains to life satisfaction (2, 3). The affective events theory suggests that an individual’s emotional state is often directly influenced by life events, with weather, as an inescapable external variable in daily life, typically representing the “atmospheric conditions” of daily living. Weather conditions can often be quantified through specific parameters such as the peak temperature or rainfall of the day (55). In addition, weather factors include humidity, duration of sunlight, forms of precipitation (e.g., rain, snow, hail), and their changes throughout the day. Existing studies indicate that weather conditions, such as temperature, sunlight, precipitation, wind speed, and air quality, significantly impact individual well-being. However, there is still relatively limited empirical research on the specific mechanisms through which weather affects well-being. Due to differences in research methods, evaluation metrics, and participant groups, the existing research results show considerable inconsistency. These differences not only suggest that the extent of weather’s impact on well-being is closely related to the specific weather conditions but also reflect the important role of individual differences in this process. Therefore, whether well-being is affected by weather conditions and to what extent depends not only on the objective weather state but also on the individual’s physiological, psychological, and social background. This paper systematically reviews and summarizes the existing research from the perspectives of theoretical foundations, direct evidence, and physiological mechanisms, and proposes corresponding theoretical support for creating favorable environmental conditions to enhance individual well-being.

## 2 Theoretical foundations of the impact of weather conditions on well-being

In the field of psychology, some scholars have applied Maslow’s Hierarchy of Needs theory to explain the impact of weather conditions on well-being (4). According to this theory, good weather conditions (such as fresh air, appropriate temperatures, etc.) fulfill an individual’s physiological and safety needs, and the satisfaction of these basic needs is an essential prerequisite for enhancing individual well-being. As theoretical and empirical research in the field of well-being continues to deepen, scholars have also proposed new theoretical frameworks to explain the mechanisms through which weather influences well-being. Environmental factors are one of several contributors to well-being, aligning with Lyubomirsky’s (5) framework. Lyubomirsky’s (5) happiness formula suggests that well-being consists of 50% genetic factors, 10% environmental factors, and 40% individual behavior. According to this formula, environmental factors play an important role in the formation of individual well-being. Specifically, when people are in pleasant external natural environments, they are more likely to experience happiness. As a key component of the natural environment, weather conditions can significantly influence an individual’s emotions and overall well-being. Weather not only affects people’s living conditions and external environmental settings but can also impact happiness by altering emotional states.

The evolutionary psychology theory further explains the relationship between weather and well-being. This theory posits that humans have an inherent positive preference for non-threatening environmental factors in nature, which helps to promote survival and enhance well-being. When individuals are in a non-threatening environment, they naturally produce positive emotional responses. These responses further influence attention, physiological reactions, and behavior, thereby enhancing well-being (6). Therefore, when weather conditions are favorable and the external natural environment is pleasant, individuals’ well-being is generally higher.

In addition, Kanazawa and Li (7), building on evolutionary psychology, proposed the “Tropical Savannah Theory of Well-being.” This theory suggests that environmental factors that contributed to the happiness of our ancestors may still have similar effects on us in modern society. Conversely, environmental factors that once diminished our ancestors’ well-being may still have negative impacts today (8). Specifically, humans, as diurnal creatures, are highly dependent on sunlight, and sunny days typically lead to higher well-being. In contrast, low light in dark environments can induce anxiety, which may be associated with potential danger signals in evolutionary history, leading to a decrease in well-being. Therefore, fundamental weather conditions such as the degree of sunlight, the variation between sunny and rainy days, and wind speed continue to influence our perception of environmental safety, comfort, and our experience of well-being.

The above theories provide profound insights into how weather influences well-being through physiological, emotional, and cognitive pathways, revealing the complex relationship between weather conditions and individual well-being.

## 3 Weather conditions as direct influences of well-being

In the field of environmental psychology, researchers typically focus on weather conditions such as light, temperature, rainfall, humidity, air quality, and wind speed. These factors are commonly used to explore how weather affects human mental health, emotional responses, behavioral patterns, and social interactions through various pathways.

Light and temperature are key factors influencing an individual’s physiological and psychological state. Ample sunlight and a comfortable temperature are often associated with positive emotions and good mental health, while gloomy, cold weather can lead to feelings of sadness and discomfort. Rainfall and humidity are closely linked to emotional fluctuations. Overcast days and high humidity environments are often accompanied by feelings of low energy and negative emotions. Air quality has a profound impact on both psychological and physiological health, especially in regions with high pollution levels, where poor air quality can lead to increased stress and mental health issues. Wind speed, as a dynamic weather factor, can also influence emotional and behavioral responses. Strong winds may induce feelings of unease or anxiety, while calm winds are more likely to contribute to psychological relaxation.

These weather conditions affect well-being by altering an individual’s physiological state, emotional experience, and interactions with the external environment. As a result, environmental psychology often considers how these multidimensional weather factors work together to shape an individual’s emotional state, social behavior, and the quality of social interactions, ultimately impacting subjective well-being.

### 3.1 Light conditions

Light conditions are a crucial factor in determining individual well-being, as they influence both emotional states and life satisfaction (9, 10). Different levels of light exposure can have varied effects on how people feel and how satisfied they are with their lives.

First of all, Light conditions can influence emotional state. Light exposure can directly impact an individual’s emotional state. Insufficient or poorly timed light can induce negative emotions, while appropriate light, particularly sunlight, tends to have a positive association on mood. Psychological studies have shown that light can act as a mood regulator. For example, research by Schwarz and Clore (11) demonstrated that people reported higher levels of happiness on sunny days compared to rainy days, reflecting the immediate impact of sunlight on emotional states. The effect of sunlight on mood is also reflected in the fact that light can either enhance or reduce feelings of joy and sadness, depending on its timing and intensity. Denissen et al. (12) found that sunshine increases both positive and negative emotions, indicating that the relationship between sunlight and mood is complex and not purely beneficial.

Secondly, light also influences overall life satisfaction. Research has shown that individuals tend to report higher life satisfaction on sunny days. For instance, Kämpfer and Mutz (13) found that respondents in Germany reported higher life satisfaction on days with clear skies, compared to more ordinary or overcast days. Similarly, studies using EU-SILC data showed that light exposure had a significant positive correlation with life satisfaction (14). The positive effect of sunlight on life satisfaction may be attributed to both its direct influence on mood and the fact that people are more likely to engage in outdoor activities when the weather is sunny, further contributing to well-being. However, studies like those by Buscha (56) have suggested that exposure to sunlight may have a negligible effect on well-being, particularly in cases where other factors, such as personal preferences or environmental stressors, influence mood more strongly.

In addition, the impact of light on both emotions and life satisfaction can be moderated by various contextual and individual factors. For example, outdoor activities often take place more frequently on sunny days, which in turn improves well-being. Additionally, exposure to sunlight may interact with personal traits like intelligence, as shown in studies examining the relationship between sunlight exposure and happiness. In these studies, higher IQ levels were associated with a reduced positive impact of sunlight on happiness (Add Health study, U.S., 1994–2008). This suggests that individual differences play a role in how sunlight affects emotional well-being.

Overall, light conditions, particularly the presence of sunlight, are important environmental factors influencing individual happiness, emotional health, and life satisfaction. Previous research into ipRGC-influenced light (IIL) responses indicates that both illuminance and correlated color temperature (CCT) affect people’s well-being (15). Moreover, illuminance and CCT can also affect occupants’ satisfaction with and comfort in the environment (16, 17), improved satisfaction with environmental conditions is associated with improved daily life satisfaction. These effects are not only direct but are also shaped by other moderating variables, such as personal behavior and contextual factors, making the relationship between light and well-being complex and multifaceted.

### 3.2 Temperature conditions

In everyday life, people often naturally associate emotional states with temperature, such as “warm love” or “chilly fear” (18). As a key weather indicator, temperature has been found to significantly impact an individual’s well-being (57). Numerous studies have established a relationship between temperature and happiness, with some finding a non-linear (U-shaped) relationship. For example, Levinson (19) conducted an analysis using data from the Integrated Social Survey (*n* = 6,035) and found an inverted U-shaped relationship between temperature and happiness, indicating that both extremely hot and cold temperatures negatively impacted well-being. Similarly, Noelke et al. (20), using data from the Gallup World Poll (*n* = 1,854,746) from 2008–2013, observed that happiness levels significantly decreased with rising temperatures. Specifically, when temperatures exceeded 70°F (21°C), individuals reported reduced positive emotions (e.g., joy, pleasure), increased negative emotions (e.g., stress, anger), and more fatigue (e.g., tiredness, lack of energy), all of which were linked to a decrease in subjective well-being. This effect was particularly pronounced in individuals with lower education levels and older age groups.

Baylis (21) used machine learning techniques to analyze sentiment from over a billion tweets on the Twitter platform and matched these reports of well-being with daily weather conditions in the users’ locations. The study found that at temperatures below 60°F, the impact of temperature on reports of well-being was minimal. While 70°F typically induced pleasant emotions, temperatures above 70°F led to a sharp decline in emotional happiness. Furthermore, significant emotional differences in happiness were found between temperature ranges of 60–70°F and 80–90°F (approximately 0.01σ), similar to the difference in emotional well-being observed between Sundays and Mondays. The study also showed that high temperatures combined with high humidity resulted in a more significant decrease in emotional happiness (21). Tsutsui (22) conducted a 516-day online diary study with 75 university students from Osaka University. The study found that hourly temperature had a stronger impact on happiness than daily average temperature. Happiness levels were highest when temperatures ranged between 10°C and 15°C (typically occurring in April and November). The study also showed that cooler temperatures helped reduce sadness and depression.

Temperature can also influence an individual’s life satisfaction. Both psychological and economic studies have shown that exposure to high temperatures tends to lower life satisfaction (23, 24). Peng et al. (25) used geospatial methods to analyze data from the International Social Survey Programme (ISSP) conducted between 2011 and 2013 across 32 countries, excluding data from Japan, Norway, and South Africa, which lacked specific interview dates. They correlated happiness (self-reported life satisfaction) with weather variables (e.g., temperature, wind speed, dew point, visibility) and geographical data. The study found a significant relationship between happiness and temperature, with people living in tropical or subtropical regions reporting lower levels of happiness compared to those in temperate or polar regions. Garcia (26) found that individual traits are crucial predictors of overall life satisfaction. In contrast, Federsen et al. (27), based on an extensive dataset of nearly 100,000 observations from Australia’s subjective well-being survey, found that temperature did not have a significant effect on life satisfaction after controlling for individual differences. This suggests that while environmental factors like temperature may influence well-being, their effects might be overshadowed by individual characteristics. Understanding the nuanced relationship between temperature and well-being can provide valuable insights into human preferences for daily thermal conditions. Such knowledge can be applied to optimize temperature settings in both personal and professional environments, ultimately enhancing comfort, well-being, and productivity.

### 3.3 Cloud cover, precipitation, humidity, and wind speed

Several weather conditions, such as cloud cover, precipitation, humidity, and wind speed, have been found to significantly affect subjective well-being, often with a negative impact on happiness.

Empirical studies have consistently demonstrated a significant negative association between subjective well-being and environmental factors such as cloud cover, precipitation, and humidity. For instance, Baylis (21) found that increased cloud cover and precipitation were associated with a decline in emotional well-being. Cuñado and De Gracia (28) used data from the European Social Survey (EES) and Spain’s Ministry of Environment, Rural and Marine Affairs (MMA) to explore the relationship between happiness and weather conditions. They found that precipitation in January had a significant negative correlation with happiness, indicating that rain was associated with lower levels of well-being. Moreover, a higher dew point (a measure of humidity) was found to correspond with lower happiness levels, reinforcing the negative relationship between air humidity and subjective well-being (23).

Wind speed has also been shown to affect life satisfaction, with the impact being more pronounced in women. Feddersen et al. (27) conducted a study using panel data and detailed weather observations to assess the effect of daily average wind speed on self-reported life satisfaction. Their fixed-effects ordinary least squares (OLS) regression analysis revealed that a one standard deviation decrease in wind speed (1.91 m/s) led to a 0.026 increase in individuals’ life satisfaction levels. This suggests that calmer wind conditions are associated with higher life satisfaction, particularly among women.

These findings highlight the complex relationship between various weather factors and well-being, suggesting that environmental conditions such as cloud cover, precipitation, humidity, and wind speed can significantly shape individuals’ emotional and psychological states, with varying impacts based on demographic factors like gender.

### 3.4 Air quality

Air quality is a critical factor influencing both the physical and mental well-being of individuals. Poor air quality, such as high levels of particulate matter (e.g., PM2.5), is closely linked to respiratory and cardiovascular diseases (58). Beyond physical health risks, air pollution also subtly damages psychological health and overall subjective well-being (SWB).

A substantial body of research shows that air quality directly impacts both individual mental health and overall happiness. For example, Li et al. (29) reviewed empirical studies on the effects of air pollution on subjective well-being, proposing the use of psychophysical methods to quantify the impact of air pollution on SWB. While there is considerable evidence of a negative correlation between air quality and happiness, some studies suggest that the relationship may not be as strong as previously thought. Zhang et al. (30), for example, used longitudinal data from a national survey and examined its association with local air quality and weather conditions. Their findings indicated a negative correlation between air pollution and hedonic well-being, as well as a positive correlation with the incidence of depression symptoms. However, no significant association was found between air pollution and life satisfaction.

Life satisfaction (LSA) is a key measure of subjective well-being and has been shown to be closely related to air pollution. Li et al. (29) found that air pollutants such as CO2, NO2, PM10, and SO2 adversely affect individuals’ perceptions of their quality of life. A review of 15 studies conducted between 2002 and 2017 found that 94% of studies reported that these pollutants negatively impacted subjective well-being. Yuan et al. (59) demonstrated a significant negative correlation between the Air Quality Index (AQI) and individuals’ life satisfaction, and Song et al. (58) found a positive correlation between urban smog levels and subjective happiness. However, this finding might be influenced by income disparities between cities.

Using an instrumental variables (IV) method, Shi and Yu (60) reassessed the relationship between air pollution and subjective well-being, confirming that PM2.5 levels are negatively correlated with happiness. This causal relationship underscores the importance of using appropriate methods to capture the impact of air pollution on subjective well-being. Hedonic methods, which focus on pleasure-based happiness measures, also show a significant negative effect of air pollution on personal happiness (61, 62).

Economists and scientists have been exploring innovative ways to examine this relationship. A recent study published in *Nature Human Behaviour* explored the emotional expressions in 210 million posts on the Chinese social media platform “Weibo” which included location markers. By matching these posts to local daily air quality data, the researchers were able to establish a real-time link between air pollution and happiness. The study, which covered data from 144 Chinese cities, found that on days with higher pollution levels, self-reported happiness was significantly lower.

These studies highlight a clear relationship between air pollution levels and happiness. While health undoubtedly plays a role, even after controlling for health status, air pollution appears to affect happiness more directly than physical health itself. The impact of air quality on happiness may be attributed to various factors, including aesthetic considerations (e.g., smog), sensory effects (e.g., smell or taste), and anxieties about personal and public health. The degree of air pollution can significantly influence individual schedules and life satisfaction, especially for vulnerable groups such as children, the older adult, and those with respiratory or cardiovascular conditions.

### 3.5 Extreme weather

Extreme weather events (EWEs) have become an increasingly important and unavoidable aspect of weather conditions. Since 1950, global temperatures have risen by 0.8°C (31), and with the ongoing global warming, the frequency and severity of extreme weather events, such as heatwaves, droughts, floods, wildfires, and hurricanes, have been on the rise (32). These events have placed a significant burden on global public health and socioeconomic systems (33). The interactions between EWEs and human health are complex and can be categorized into two main types: (1) direct impacts, which result from extreme physical climate phenomena such as storms or floods, and (2) indirect impacts, which typically arise from environmental changes, such as water quality deterioration or land use changes, that affect biogeophysical processes (63, 64). These impacts interact with social factors (e.g., demographics) and may modify (amplify or mitigate) the intensity of subsequent effects, ultimately influencing mental health and well-being (63).

As a category of extreme weather, hurricanes have a significant negative impact on individuals’ emotional well-being. For instance, Hurricane Maria in 2017 caused massive devastation in Puerto Rico. A study involving 226,808 Puerto Rican adolescents revealed that 5 to 9 months after the hurricane, participants reported increased levels of negative emotions such as depression (65). Similarly, following Hurricane Katrina, a study of 325 adolescents who experienced the storm found that participants generally showed signs of anxiety and other negative emotions (66). Wildfires, like other extreme weather events, also have a detrimental effect on emotional well-being. Studies have found that people who have experienced wildfires show increased incidences of anxiety, depression, and other mental health problems.

Additionally, research indicates that an individual’s past experiences of damage caused by extreme weather events, coupled with expectations about future climate change, can jointly lead to a negative impact on current subjective well-being (34). The emotional toll from such events is not only a response to the immediate effects but also a product of anticipation and uncertainty regarding future environmental challenges. This highlights the significant role that climate events play in shaping long-term psychological well-being, especially as individuals and communities face an increasingly unpredictable climate.

### 3.6 Interaction of different weather conditions

Since different weather variables are often interconnected, it is essential to consider a comprehensive view of various weather factors. Weather changes not only influence the generation and degradation of pollutants in the air but also lead to the cross-regional transfer of these pollutants. Air quality is a result of both natural processes and human emissions into the atmosphere, and it is also the outcome of complex physical and chemical reactions of many substances in the air. Therefore, without considering weather conditions, researchers cannot accurately predict air quality. By incorporating factors such as atmospheric pressure, precipitation, temperature, humidity, wind speed, and sunlight, we can better understand the causes and trends of daily air quality variations (12).

In general, favorable weather conditions (e.g., sunny, warm, low humidity, and gentle breezes) encourage people to engage in outdoor activities, but these conditions also create conditions that favor the generation and stagnation of air pollution. For example, sunshine and high temperatures not only contribute to the generation of particulate matter in the air but also facilitate the formation of ozone. In contrast, weather conditions that make people want to stay indoors (e.g., precipitation, low temperatures, and strong winds) are more conducive to fresh air and fewer pollutants in the atmosphere. Research has shown that powerful typhoons and rainfall can purify polluted air, thus improving air quality (25).

Ground-level ozone and particulate matter are the two main pollutants reported in air quality index forecasts. Ground-level ozone is formed due to two key pollutants—NOx (nitrogen oxides) and VOCs (volatile organic compounds)—which react under sunlight during warm weather. Ozone, formed during the day, shows a relatively stable pattern, but it disappears slowly after sunset. Ozone can also be transported by wind from one area to another, leading to pollution not only in urban centers but also in surrounding suburban and rural areas. On the other hand, particulate pollution can be directly emitted (e.g., from smokestacks) or formed through gas reactions or interactions with other aerosols in the atmosphere. Nitrogen oxides and sulfur dioxide contribute to the formation of particulate matter. The sources of these ozone and particulate pollutants are diverse, including automobiles, power plants, and factories. Wildfires and volcanoes also produce particulate matter, and trees and other vegetation contribute to both particulate and ozone pollution.

Furthermore, the impact of weather conditions on subjective well-being (SWB) is moderated by variables such as gender, age, and socioeconomic status (SES). First, research shows that the effect of weather conditions on individual happiness differs significantly by gender. For instance, a 6-week diary study by Barnston (35) found that men’s emotions were more affected by weather than women’s. Men were more sensitive to temperature changes and preferred cooler weather, while women’s happiness was less influenced by temperature variations. This indicates that individual characteristics, such as gender, can influence the extent to which weather affects well-being, highlighting the complexity of the relationship between weather and happiness.

Overall, the interplay between weather conditions and subjective well-being is multifaceted, involving both direct and indirect effects that vary across individuals and contexts. The influence of weather on emotional health and life satisfaction is shaped not only by the specific weather conditions but also by personal and social factors.

## 4 The physiological mechanisms through which weather conditions indirectly affect happiness

Weather conditions not only directly influence an individual’s level of happiness but can also impact happiness by causing physiological changes.

### 4.1 Melatonin

Melatonin is an indole hormone synthesized in the pineal gland, and its production is significantly influenced by environmental light conditions. This hormone is synthesized from serotonin via the tryptophan-5-hydroxytryptamine biosynthesis pathway, and its secretion is related to the input from the brain’s circadian rhythm center. As ambient light decreases, melatonin secretion increases, reaching its peak in darkness, which promotes feelings of fatigue and sleepiness. Conversely, when light levels increase, the presence of light suppresses melatonin production, leading to a reduction in circulating melatonin levels in the plasma. This photo sensitivity characteristic plays a key role in regulating the human sleep–wake cycle.

The impact of melatonin on happiness can be seen through its regulation of neurochemicals. Studies have shown that melatonin influences the production and release of neurochemicals such as serotonin, dopamine, and endorphins, which are associated with happiness and well-being (36). Melatonin acts like the conductor of an orchestra, integrating various neurochemical elements to create a beautiful “symphony,” thus maintaining our smiles and sense of satisfaction. However, lifestyle choices and habits can interfere with melatonin production, which in turn affects this delicate balance. For example, artificial light at night, caffeine and alcohol consumption, and high stress levels can all impact melatonin production, affecting our happiness. These findings highlight melatonin’s potential role in regulating mood and well-being, suggesting that melatonin is not only a hormone that regulates sleep but may also play a role in broader psychological health and emotional regulation.

### 4.2 Vitamin D

Weather conditions can have a significant impact on vitamin D synthesis. Vitamin D is primarily synthesized in the skin under sunlight, especially through ultraviolet B (UVB) exposure. Therefore, cloudy days, winter, or any weather conditions that reduce sunlight can decrease the natural synthesis efficiency of vitamin D. On cloudy or foggy days, the intensity of UV rays weakens, which may reduce the skin’s ability to produce vitamin D. Additionally, in winter, due to changes in the angle of solar radiation and reduced daylight hours, UVB radiation intensity decreases, further reducing opportunities for skin to synthesize vitamin D.

The benefits of vitamin D for mental health and happiness have been confirmed in multiple studies. For example, a meta-analysis by Głąbska et al. (37) found a significant association between vitamin D levels and mood. Vitamin D deficiency can lead to depressive feelings and fatigue. The absence of vitamin D is linked to depressive symptoms and negative emotions. Some studies have shown that low blood levels of vitamin D are associated with increased depressive symptoms. Individuals who have attempted suicide tend to have much lower vitamin D levels than other depressed patients who have not attempted suicide or healthy control groups. Supplementing vitamin D has been shown to reduce certain types of inflammation related to suicide. A study by Cereda et al. (38) pointed out that in winter, reduced sunlight exposure and lower vitamin D levels may induce depressive symptoms in individuals with Seasonal Affective Disorder (SAD), as well as conditions like bipolar disorder. Alcubierre et al. (39) found that vitamin D deficiency in diabetic patients was associated with self-reported life satisfaction. These studies show that vitamin D is not only crucial for physical health but also plays an important role in maintaining and promoting psychological well-being.

### 4.3 Serotonin

Serotonin (also known as 5-hydroxytryptamine or 5-HT) is a neurotransmitter that regulates mood and is highly sensitive to weather changes and light levels. Serotonin is primarily located in the central nervous system, gastrointestinal mucosa, and platelets (40). When serotonin is released into the synaptic cleft, it may interact with both pre-synaptic and post-synaptic receptors. Studies have shown that exposure to light activates serotonin synthesis in yeast extract, indicating a direct relationship between sunlight and serotonin production (41). Additionally, light influences serotonin binding at serotonin 1A receptor sites, with lower light levels associated with lower binding levels in the cortical and subcortical regions of the brain (42). Lambert et al. (43) sampled jugular vein blood from 101 healthy male volunteers over 12 months and found that serotonin levels were lowest during the winter when there was the least sunlight.

Seasonal Affective Disorder (SAD) is a recurrent form of depression that commonly occurs in the fall and winter, often referred to as “winter depression.” Research has shown that SAD is related to shorter exposure to sunlight (67). When spring arrives and sunlight increases, symptoms tend to improve. It should be noted that studies across different populations have found significant gender and age differences in SAD symptoms (44, 45), SAD is more prevalent among individuals with a predisposition for deep depression. These research findings collectively suggest that normal adult serotonin levels are influenced by sunlight. When light decreases, serotonin secretion is reduced, which may lead to the onset of negative emotional states.

## 5 Coping strategies

### 5.1 Increasing light exposure

First, exposure to sunlight has numerous benefits for improving individual happiness and life satisfaction. Feddersen et al. (27) found that if daily sun exposure exceeds the average by one standard deviation (6.43 MJ/m^2^), an individual’s life satisfaction score (on a scale from 0 to 10) increases by 0.012. Second, exposure to sunlight increases the levels of vitamin D and serotonin, both of which enhance emotional well-being (46). Although any amount of outdoor light helps boost serotonin levels, morning sunlight seems to have the most significant effect. Some studies have found that sunbathing can help alleviate symptoms of Seasonal Affective Disorder (SAD) (47). Therefore, when weather conditions and time permit, individuals can opt for more outdoor sun exposure. When staying indoors or working in an office, sitting by south-facing windows as much as possible can also help. Third, when sunlight is insufficient, artificial light can be used to create optimal lighting conditions. For instance, full-spectrum light bulbs, which emit light similar to natural sunlight, can improve well-being. Replacing regular light bulbs with full-spectrum bulbs can positively impact an individual’s happiness. Based on this, light therapy has also been proven to be an effective treatment for depression, particularly for conditions like Seasonal Affective Disorder (48). Additionally, many empirical studies have examined the interaction between illuminance, spectrum, and subjective mood (49, 50). Fourth, when unwilling to sunbathe, browsing images containing sunlight can also have benefits. Although most buildings have windows, ensuring that the indoor environment receives adequate sunlight, many people tend to avoid exposure to the sun. Research has shown that browsing advertisements containing sunlight imagery can have a positive impact on individual emotions (51).

### 5.2 Exercise

Studies show that regular exercise can help prevent Seasonal Affective Disorder (SAD). Exercise boosts serotonin and endorphin levels, both of which induce a “runner’s high,” and this euphoric feeling from running has been shown to combat depression (52). Experimental results from Siebers et al. (31) found that compared to walkers, individuals who ran on a treadmill for 45 min showed increased feelings of euphoria and decreased anxiety. Therefore, moderate exercises such as running are excellent choices for enhancing positive emotions. Specifically, when the weather is good, engaging in physical activities outdoors or in well-lit indoor areas makes the positive effects even more pronounced. Taking a moderate outdoor exercise break during lunch hours significantly benefits an individual’s happiness. However, when air pollution levels are high, people should reduce or avoid outdoor physical activities as a preventive and protective measure.

### 5.3 Diet

Diet is another essential way for individuals to obtain vitamin D, aside from sunlight (53). In late autumn and winter, when sunlight-induced vitamin D production is low, individuals can increase their intake of vitamin D-rich foods such as egg yolks, soy milk, mushrooms, cheese, animal liver, skim milk, and seafood (54). The Vitamin D Council recommends a daily intake of 2000 IU of vitamin D, with higher supplementation for those who do not get enough sun exposure. Additionally, consuming fatty fish, such as salmon and sardines, which contain omega-3 fatty acids, is beneficial for reducing negative emotions. Research has shown that a deficiency in eicosapentaenoic acid (EPA) and docosahexaenoic acid (DHA) increases the risk of depression, and eating fish can effectively replenish these two essential fatty acids. Furthermore, eating fresh fruits and vegetables not only benefits physical health but also has a significant positive correlation with happiness (25). Lastly, although melatonin is primarily synthesized by the body, some individuals may lack the ability to produce or release melatonin properly. In such cases, taking melatonin supplements is relatively safe and does not lead to dependency, making the risk of misuse quite low.

## 6 Conclusion

This study begins by exploring the theoretical foundations of environmental psychology and bioclimatology, explaining how weather influences human psychological states and behaviors. It then analyzes the direct effects of weather conditions on happiness, specifically discussing how sunlight, temperature, humidity, and other weather factors directly impact people’s emotions and well-being, and how these effects vary due to individual differences. Subsequently, the physiological mechanisms through which weather conditions affect happiness are explained. For example, sunlight affects the production of melatonin and vitamin D, thereby influencing mood and mental health.

Currently, although the impact of weather on happiness has been supported by both theoretical and empirical research, there are still many uncertainties in these findings, which create challenges in creating conditions that enhance happiness for individuals. First, researchers have yet to reach a consensus on the definition of “good weather” (68). Correspondingly, people’s preferences for weather in daily life also vary. “What is sweet for one is bitter for another”—some people prefer sunny days, while others enjoy rainy weather. These preferences seem to present an irreconcilable contradiction, making it paradoxical to identify a single “good weather” that caters to everyone’s happiness. Second, different weather factors are often correlated, making it difficult to isolate the effect of a single weather factor on happiness without the interference of other variables. As Denissen et al. (12) pointed out, focusing solely on a single weather parameter might fail to identify the specific effects of certain weather features. Third, even for the same individual, the emotional experience brought about by the same weather condition can vary significantly depending on the time or situation. Additionally, the effect of weather conditions on happiness can be influenced by the activities individuals engage in on a given day, and the impact of those activities may mask the role of weather conditions. Finally, there is a lack of experimental research on the relationship between weather conditions and happiness, which prevents us from concluding whether there is a causal relationship between the two.

In conclusion, exploring the relationship between weather conditions and happiness, and finding strategies to enhance happiness, remains an emerging and evolving research topic that requires collaboration from researchers across various disciplines.

## 7 Future research directions

To advance the understanding of the relationship between weather conditions and well-being, future research should prioritize longitudinal and experimental studies. Longitudinal studies would allow researchers to track changes in well-being over time in response to varying weather conditions, thereby providing stronger evidence for potential causal links. Experimental studies, on the other hand, could help isolate the specific effects of different weather variables by controlling for confounding factors in a controlled setting.

Additionally, an interdisciplinary approach is essential for a more comprehensive exploration of this complex relationship. Integrating perspectives from environmental psychology, climatology, and behavioral sciences can provide a deeper understanding of the underlying mechanisms that connect weather conditions to well-being. Environmental psychology can offer insights into perceptual and cognitive processes related to weather experiences, while climatology can contribute expertise on atmospheric patterns and long-term climate trends. Behavioral sciences, including psychology and sociology, can help elucidate how individual differences and social contexts mediate the impact of weather on well-being.

By combining these methodological and interdisciplinary perspectives, future research can develop more robust models to explain how weather conditions influence well-being, ultimately informing policies and interventions aimed at optimizing environmental conditions for improved mental health and life satisfaction.
